# Assessment of myocardial perfusion using dual-source computed tomography myocardial perfusion imaging in a goat model of reperfusion injury induced by distal microembolization

**DOI:** 10.3389/fcvm.2026.1809725

**Published:** 2026-10-14

**Authors:** Hui Jin, Chaowei Duan, Xiaolin Chen, Yaojun Lu, Xiaolin Liu, Meng Li

**Affiliations:** 1 Department of Cardiology, Baotou Central Hospital, Baotou, Inner Mongolia, China; 2 Department of Radiology, Baotou Central Hospital, Baotou, Inner Mongolia, China

**Keywords:** DSCT-MPI, goat model, microspheres, microvascular obstruction, myocardial ischemia-reperfusion injury, myocardial perfusion imaging

## Abstract

**Background:**

Myocardial ischemia–reperfusion injury (MIRI) contributes to adverse outcomes after acute myocardial infarction. Microvascular obstruction (MVO), a key component of MIRI, impairs myocardial perfusion despite successful epicardial revascularization. This study aimed to establish a reproducible large-animal model of embolization-induced MVO and evaluate myocardial perfusion heterogeneity using dual-source CT myocardial perfusion imaging (DSCT-MPI).

**Methods:**

Eight male Saanen goats underwent 30 min of left circumflex artery occlusion followed by distal microcatheter injection of 20-*μ*m polystyrene microspheres to induce reperfusion-associated MVO. DSCT-MPI was performed immediately after model establishment, with myocardial perfusion assessed by iodine distribution. Pathology was evaluated using triphenyltetrazolium chloride and hematoxylin–eosin staining, and Bcl-2, Bax, and caspase-3 were quantified. Receiver operating characteristic (ROC) analysis was used to determine the iodine concentration threshold for infarcted myocardium.

**Results:**

Seven of eight goats survived and underwent DSCT-MPI. Of 119 myocardial segments, 39 were infarcted, 14 were non-infarcted hypoperfused segments consistent with distal MVO, and 66 were normal. Binary ROC and diagnostic-performance analyses included the 39 infarcted and 66 normal segments, excluding the 14 MVO-consistent segments. At an iodine concentration cutoff of 1,582 μg/cm^3^, DSCT-MPI achieved 89.7% sensitivity, 90.9% specificity, and an AUC of 0.848 (95% CI, 0.736–0.959), with substantial histopathological agreement (*κ* = 0.798). Infarcted myocardium showed reduced Bcl-2 and increased Bax and caspase-3 expression. The 14 hypoperfused segments without TTC-confirmed necrosis were classified as MVO-consistent rather than CMD.

**Conclusions:**

A minimally invasive, reproducible goat model of embolization-induced myocardial reperfusion injury was established. DSCT-MPI identified infarcted myocardium and non-infarcted hypoperfused regions consistent with distal MVO, demonstrating post-reperfusion perfusion heterogeneity without supporting CMD. Because no direct CMR comparison was performed, these findings should be considered exploratory pathology-based validation.

## 1 Introduction

Microvascular obstruction (MVO) is a key pathological determinant of impaired myocardial perfusion following reperfusion therapy in acute myocardial infarction, despite restoration of epicardial coronary flow (1). Unlike coronary microvascular dysfunction (CMD), which primarily reflects functional or structural abnormalities of the microcirculation in the absence of obstructive coronary artery disease, MVO is most commonly caused by distal embolization, endothelial injury, and capillary destruction occurring during reperfusion (2–5). Accordingly, CMD and MVO represent distinct pathophysiological entities and should not be used interchangeably in the present study.

In clinical settings, embolization of thrombotic or atheromatous debris during percutaneous coronary intervention (PCI) is a common mechanism underlying MVO (6). To better characterize this phenomenon, experimental models that reproduce embolization-induced microvascular obstruction are required. Although CMD and MVO are sometimes collectively described as “microcirculatory dysfunction,” they differ fundamentally in pathophysiology, clinical context, and diagnostic criteria (7). The present study, therefore, focuses specifically on embolization-induced MVO rather than functional CMD, and all hypoperfused but non-infarcted myocardial segments are described accordingly.

Dual-source computed tomography myocardial perfusion imaging (DSCT-MPI) has emerged as a non-invasive imaging technique for evaluating myocardial perfusion by quantifying iodine distribution within the myocardium. DSCT-MPI has been primarily applied to identify infarcted or ischemic myocardium; however, regional perfusion heterogeneity detected after reperfusion may reflect the presence of distal microvascular obstruction rather than direct evidence of CMD. Accordingly, this study does not aim to diagnose CMD using DSCT-MPI, and DSCT-MPI findings are interpreted only in relation to infarction and MVO-consistent hypoperfusion.

Myocardial ischemia–reperfusion injury (MIRI) refers to myocardial damage that occurs after restoration of coronary blood flow and encompasses multiple mechanisms, among which MVO plays a central role. If a stable large-animal MIRI model incorporating distal embolization can be established, it may provide objective experimental data for translational studies of reperfusion injury. Previous animal models have largely relied on surgical coronary artery ligation in small animals, which is technically demanding, associated with high perioperative mortality, and limited in clinical translatability. Large-animal models using dogs or pigs more closely resemble human coronary anatomy but are costly and difficult to maintain (8). Goats offer practical advantages, including manageable size, lower cost, and coronary anatomical characteristics comparable to humans, with relatively limited collateral circulation (9–11).

In the present study, the left circumflex artery (LCX) was selected for model construction to ensure technical feasibility, procedural reproducibility, and avoidance of extensive lethal infarction that may occur with left anterior descending artery occlusion. While this approach allows consistent induction of regional ischemia followed by distal embolization, adaptation of this methodology to larger myocardial territories is feasible but was not investigated in the current study. Saanen goats underwent PCI-based transient coronary occlusion followed by distal microcatheter injection of 20-μm polystyrene microspheres to simulate post-reperfusion microvascular embolization.

This study aimed to establish a reproducible large-animal model of ischemia–reperfusion injury complicated by distal microvascular obstruction and to evaluate myocardial perfusion heterogeneity using DSCT-MPI. Although the model does not recapitulate CMD as a distinct pathological entity, the identification of non-infarcted hypoperfused regions reflects key pathophysiological features of embolization-induced MVO.

## 2 Materials and methods

### 2.1 Animals

Eight male Saanen goats aged 5–8 months and weighing 26.5 ± 5.1 kg were used in this experiment. The goats were purchased from Xi'an Zhenping Bio Technology Co. All animal experiments were conducted in accordance with the Guide for the Care and Use of Laboratory Animals and were approved by the Ethics Committee of Baotou Central Hospital.

### 2.2 Experimental methods

#### 2.2.1 Laboratory animal preparation and anesthesia

Goats were fasted for 24 h before surgery, and water was withheld for 8 h. Before auricular vein cannulation, sedation was achieved with xylazine (0.05–0.10 mg/kg, intramuscularly). An indwelling catheter was then placed in the auricular vein and connected to a three-way stopcock for intravenous access. Anesthesia was induced with propofol (2.5–3.5 mg/kg, intravenous) and maintained at 22–30 mg/kg/h as required. Throughout the procedure, physiological status was continuously monitored using a 12-lead electrocardiogram and pulse oxygen saturation monitor, and an external defibrillator was kept available.

#### 2.2.2 Animal model preparation

After anesthesia induction, the goat was positioned supine, routinely disinfected, and draped. Using the Seldinger technique, the right femoral artery was cannulated and a 6-F arterial sheath was inserted. Antithrombotic medication consisted of heparin (100 U/kg, intra-arterially through the sheath immediately after sheath placement). Antiarrhythmic medication was not routinely administered prophylactically but was reserved for rescue treatment if malignant ventricular arrhythmias occurred. Under fluoroscopic guidance with a Philips Allura Xper FD20 angiographic system, a 6-F EBU guiding catheter was advanced to the left coronary ostium for coronary angiography. A 0.014-inch BMW guidewire was then placed in the LCX, and an appropriately sized balloon catheter was inflated to occlude the LCX for 30 min to induce regional myocardial ischemia. After ischemia, a 1.7-F microcatheter was advanced distal to the balloon occlusion, and 20-μm polystyrene monodisperse microspheres (Thermo Fisher Scientific, USA) were slowly injected to produce distal microvascular obstruction during reperfusion (Figure 1). The embolic suspension consisted of 1 mL microsphere stock solution (approximately 5.8 × 10⁶ microspheres) diluted with 9 mL nonionic contrast medium (iohexol), administered in divided boluses followed by a gentle saline flush. This procedure was designed to reproduce myocardial ischemia–reperfusion injury complicated by distal microvascular obstruction rather than isolated coronary microvascular dysfunction.

**Figure 1:**
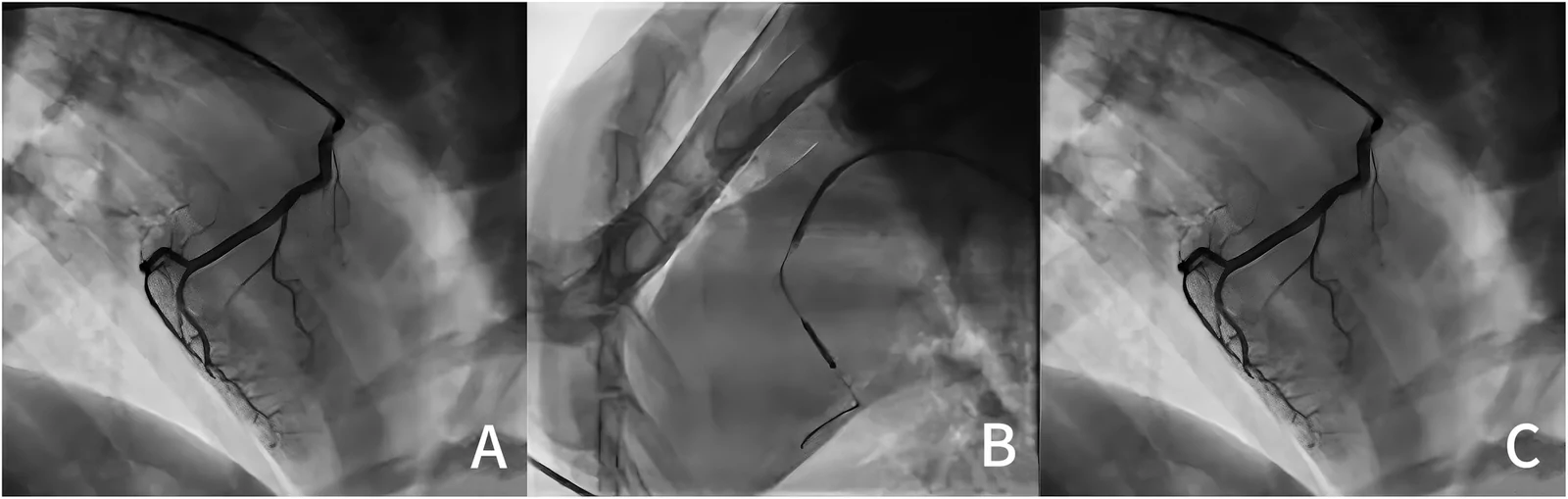
Coronary angiography images demonstrating the key interventional steps used to establish the goat myocardial ischemia–reperfusion injury model with distal microvascular obstruction. **(A)** Baseline coronary angiography showing normal antegrade flow in the left circumflex artery. **(B)** Balloon occlusion of the left circumflex artery with absence of distal contrast flow. **(C)** Post-embolization angiogram demonstrating impaired distal perfusion following microsphere injection.

#### 2.2.3 Electrocardiographic monitoring

ECGs were recorded before balloon occlusion, at 30 min of ischemia, and at 2 h after reperfusion. ST-segment elevation in at least two leads (≥0.2 mV in precordial leads or ≥0.1 mV in limb leads) was considered indicative of acute myocardial ischemic injury. ST-segment resolution ≤30% after reperfusion was interpreted as evidence of persistent microvascular perfusion impairment, supporting successful model establishment.

#### 2.2.4 Dual-source computed tomography myocardial perfusion imaging (DSCT-MPI)

The DSCT-MPI was conducted right after establishing the model via a third-generation dual-source computed tomography (CT) system (Siemens Definition Force, Forchheim, Germany). An iodine-based contrast agent, iohexol (1.5 mL/kg), was injected intravenously at a rate of 3.0 mL/sec utilizing a power injector, which was then immediately followed by an additional 35 mL of saline. A region-of-interest threshold of 100 HU was triggered, and scan initiation began at 7 s after peak temporal enhancement.

A solid-state digital workstation developed by Siemens was used to reconstruct the images, after which the iodine distribution maps from the two trained readers (who did not know the pathological results) were independently examined. Inter-reader agreement between the two blinded observers was excellent, with an intraclass correlation coefficient (ICC) of 0.88 (95% CI: 0.81–0.93) for quantitative iodine concentration measurement and a Cohen's kappa of 0.82 for categorical perfusion classification. The individual myocardial segments were categorized visually as being hyper, hypo, or non; however, these were further quantitatively analyzed by calculating the individual segment's iodine concentration (in micrograms per cubic centimeter). If any myocardial segment had an iodine concentration decrease of greater than 20% from neighbouring normal myocardial segments, it was defined as experiencing significant hypoperfusion.

#### 2.2.5 Myocardial segmentation and definition of perfusion abnormalities

According to the 17-segment model of myocardial segmentation approved by the American Heart Association, myocardial segmentations were determined based upon the criteria described below in each category noted:

- Infarcted Segments: segments that display hypoperfusion as determined via DSCT-MPI and, through TTC staining, demonstrate evidence of cardiac muscle necrosis.
- Non-infarcted Hypoperfused Segments Consistent with Distal MVO: separate from the infarcted muscle, significant hypoperfusion was observed via the DSCT-MPI, and the absence of any evidence of cardiac muscle necrosis on TTC staining.
- Normal Segments: as assessed by both the DSCT-MPI, which demonstrates normal perfusion patterns, and the results of the TTC staining, no stains demonstrating the presence of cellular necrosis exist.

No myocardial segment in the present study was classified as isolated CMD; all non-necrotic hypoperfused segments were categorized as MVO-consistent according to the imaging-pathology framework.

During the perioperative period, antithrombotic therapy consisted of heparin (100 U/kg, administered intra-arterially through the arterial sheath immediately after sheath placement). Antiarrhythmic drugs were not routinely administered prophylactically but were reserved for rescue treatment in the event of malignant ventricular arrhythmias.

### 2.3 Pathology

After model establishment, the animals were recovered and observed for 1 week. Postoperative analgesia was provided with flunixin meglumine (1.1 mg/kg, intramuscularly, once daily for up to 3 days, as needed). At 1 week, the animals were euthanized by intravenous pentobarbital sodium overdose (100 mg/kg). The hearts were then rapidly excised, and the ventricles were cut into transverse slices (approximately 20 mm thick) from apex to base. The slices were stained with 2,3,5-triphenyltetrazolium chloride (TTC) to distinguish viable from infarcted myocardium. Tissue samples from the LCX perfusion territory were fixed in 4% paraformaldehyde, embedded in paraffin, sectioned at 2 μm, and stained with hematoxylin–eosin for histopathological examination.

### 2.4 Apoptosis-related indicators

Myocardial tissue homogenates from normal and infarcted regions were prepared to quantify Bcl-2-associated X protein (Bax), B-cell lymphoma-2 (Bcl-2), and caspase-3 using enzyme-linked immunosorbent assay kits (Bax ELISA kit: Abcam, Cambridge, UK; catalog No. ab199080; Bcl-2 ELISA kit: Abcam, catalog No. ab119506; caspase-3 ELISA kit: Abcam, catalog No. ab181418), following the manufacturers’ instructions.

### 2.5 Outcome measures

Histopathological assessment was used as the reference standard for evaluating the diagnostic performance of DSCT-MPI in detecting infarcted myocardial segments. Sensitivity, specificity, positive predictive value, negative predictive value, overall accuracy, and Cohen's kappa were calculated using only infarcted and normal myocardial segments. Non-infarcted hypoperfused segments consistent with distal MVO were analyzed descriptively as a separate category and were excluded from the binary diagnostic-performance analysis.

The formula definitions were omitted from this report because of the nature of these metrics being well-known and accepted within the medical community.

### 2.6 Statistical methods

SPSS version 22.0 (SPSS Inc, Chicago, IL, USA) was used for statistical analyses. Continuous variables were expressed as mean ± standard deviation. Normality was assessed using the Kolmogorov–Smirnov test. For comparisons between normal and infarcted myocardium at the animal level, paired t-tests or Wilcoxon signed-rank tests were used as appropriate. Because multiple myocardial segments were obtained from the same goat, segment-level observations were not treated as fully independent. Repeated-measures data were therefore analyzed using a generalized estimating equation (GEE) approach with goat as the clustering variable to account for within-animal correlation. Receiver operating characteristic (ROC) analysis was performed at the myocardial segment level to identify the optimal iodine concentration threshold for infarcted myocardium. The ROC analysis included 39 infarcted and 66 normal myocardial segments from seven animals. Fourteen non-infarcted hypoperfused segments consistent with distal MVO were excluded from the binary ROC analysis because they represented a separate imaging–pathology category rather than unequivocally normal myocardium. Animal-level ROC analysis was not performed. Because the myocardial segments were clustered within animals, the number of animals, rather than the total number of segments, was considered the primary determinant of statistical precision. Given the exploratory nature of the study, statistical uncertainty was assessed primarily from the width of the 95% confidence interval of the AUC rather than from a conventional *post-hoc* power calculation, which could overestimate power if myocardial segments were incorrectly treated as independent observations. An approximate sample-size projection for a future confirmatory study was derived using the inverse-square relationship between confidence-interval width and sample size, assuming a similar effect magnitude, segment distribution, and within-animal correlation. Agreement between DSCT-MPI and histopathological assessment of myocardial infarction was evaluated using kappa statistics. A two-sided *P* value < 0.05 was considered statistically significant.

Inter-reader agreement for DSCT-MPI image interpretation was assessed using intraclass correlation coefficient (ICC) for continuous iodine measurements and Cohen’s kappa for categorical assessment.

## 3 Results

### 3.1 Changes in body surface ECG

Based on the findings from goat experiments, acute myocardial infarction (AMI) and subsequent reperfusion appear to impact the electrocardiogram (ECG) of goats similarly to humans. The initial ECG changes after coronary artery occlusion included an elevation of T-waves and ST-segment elevation, and partial (≤30%) resolution of ST-segment elevation after reperfusion. Occasional premature ventricular and atrial contractions were observed during model establishment. One goat died during ischemia–reperfusion due to ventricular fibrillation that could not be successfully reversed. The remaining seven goats survived to the planned endpoint without sustained malignant arrhythmias (Figure 2).

**Figure 2:**
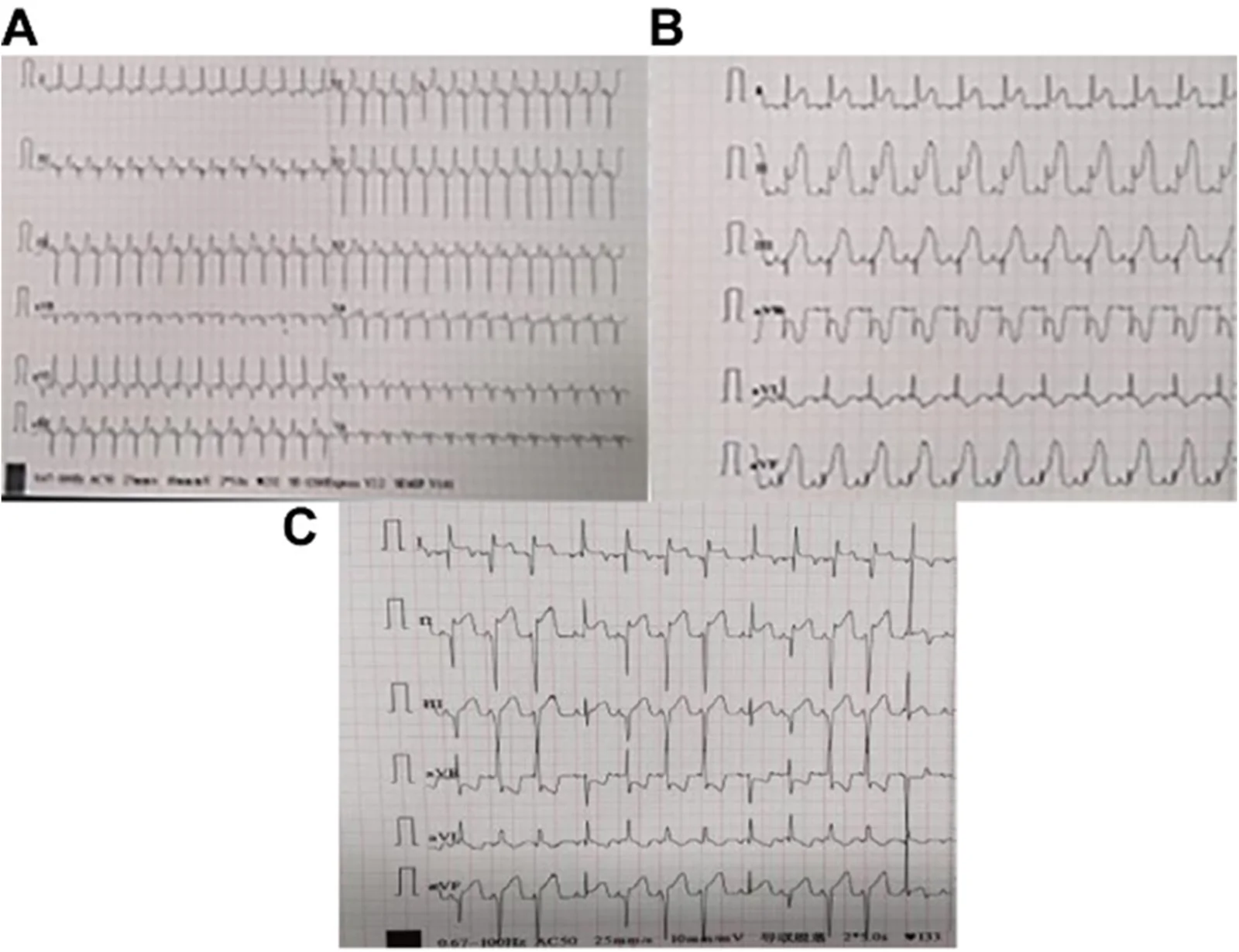
Representative electrocardiogram (ECG) changes during different phases of myocardial ischemia–reperfusion injury in goats. **(A)** Baseline ECG showing normal sinus rhythm. **(B)** ECG recorded during coronary occlusion demonstrating ST-segment elevation in leads II, III, and aVF. **(C)** ECG obtained after reperfusion showing Q-wave development and T-wave inversion.

### 3.2 Expression of apoptosis-related markers and iodine concentration in myocardium

Compared with normal myocardium, infarcted myocardium showed significant differences in apoptosis-related protein expression and iodine concentration (Table 1). Bcl-2 expression was significantly lower in infarcted myocardium (52.20 ± 2.67) than in normal myocardium (96.93 ± 1.45, *P* < 0.01). Bax (128.06 ± 3.64 vs. 47.53 ± 1.89, *P* < 0.01) and caspase-3 (123.16 ± 5.38 vs. 29.44 ± 1.26, *P* < 0.01) levels were significantly higher in infarcted myocardium. The iodine concentration was also significantly lower in infarcted myocardium than in normal myocardium (1.17 ± 0.04 vs. 3.47 ± 0.10 mg/mL, *P* < 0.01).

**Table 1:** Bcl-2, Bax, caspase-3, and iodine concentration in normal and infarcted myocardium.

Group	Bcl-2	Bax	caspase-3	Iodine concentration (mg/mL)
Normal myocardial	96.93 ± 1.45	47.53 ± 1.89	29.44 ± 1.26	3.470 ± 0.97
Infarcted myocardium	52.20 ± 2.67*	128.06 ± 3.64*	123.16 ± 5.38*	1.169 ± 0.417*
t value	6.094	2.423	11.653	4.872
*P* value	<0.001	<0.001	<0.001	<0.001

Compared with the normal myocardium group. *P < 0.01.

### 3.3 Imaging studies

Myocardial perfusion heterogeneity was demonstrated by DSCT-MPI after model establishment. The iodine maps showed decreased iodine concentration in the lateral wall myocardium, which was qualitatively identified as a hypoperfused region relative to adjacent normally perfused myocardium (Figures 3A,B). These findings were consistent with regional perfusion heterogeneity following reperfusion injury.

**Figure 3:**
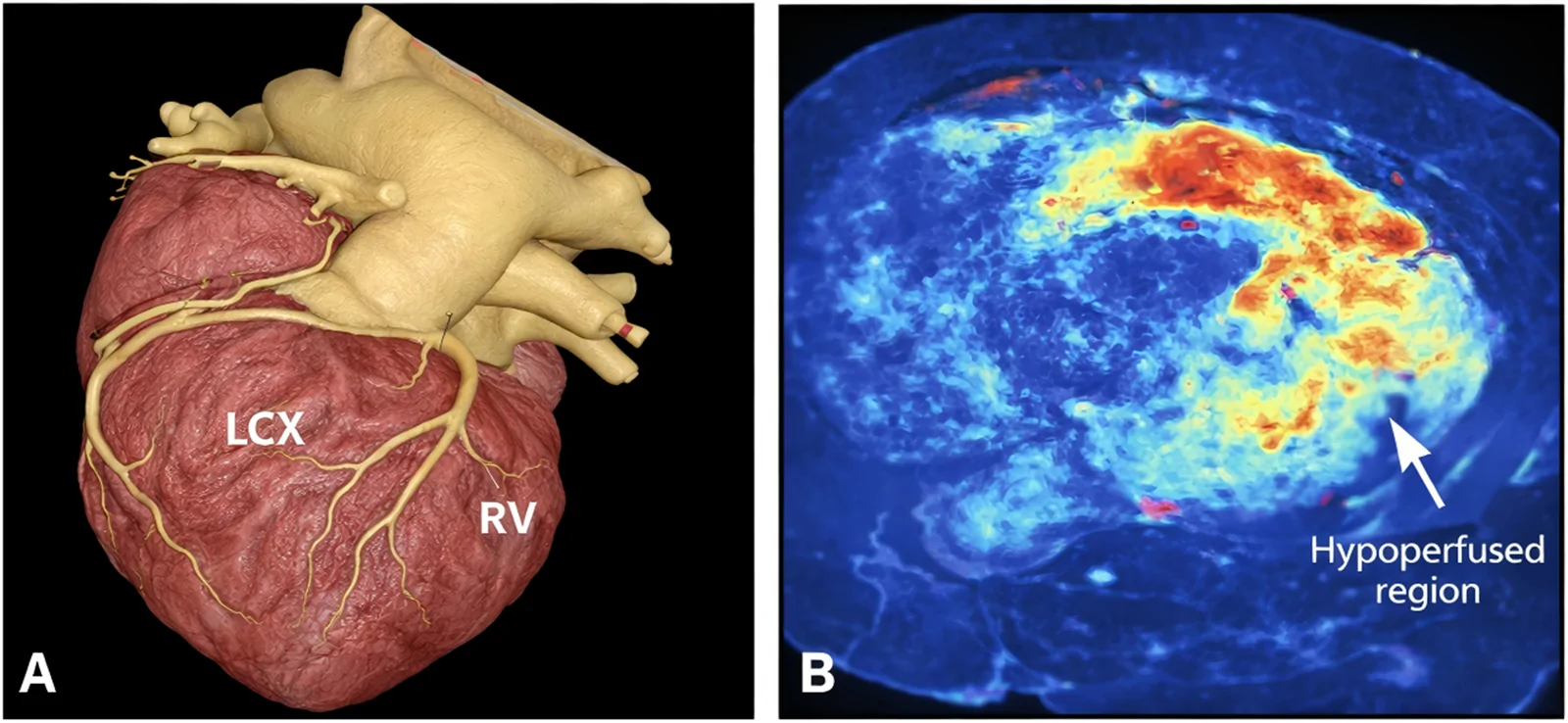
Dual-source computed tomography myocardial perfusion imaging (DSCT-MPI) demonstrating regional myocardial hypoperfusion in the goat ischemia–reperfusion model. **(A)** Three-dimensional volume-rendered image showing cardiac anatomy. **(B)** Axial iodine distribution map demonstrating a hypoperfused region in the lateral wall myocardium (arrow), with reduced iodine concentration compared with adjacent normally perfused myocardium.

### 3.4 Histopathology

According to the gross morphology of the left ventricular and inferior myocardial walls, infarction was observed in all seven goats that survived to the end of the experiment. The normal myocardium was bright red on gross examination, whereas necrotic regions appeared grayish white in the inferior wall and apex of the left ventricle. Hematoxylin–eosin (H&E) staining demonstrated normal myocytes in noninfarcted regions and necrotic cardiomyocytes with inflammatory cell infiltration, interstitial edema, and nuclear pyknosis in infarcted regions (Figure 4).

**Figure 4:**
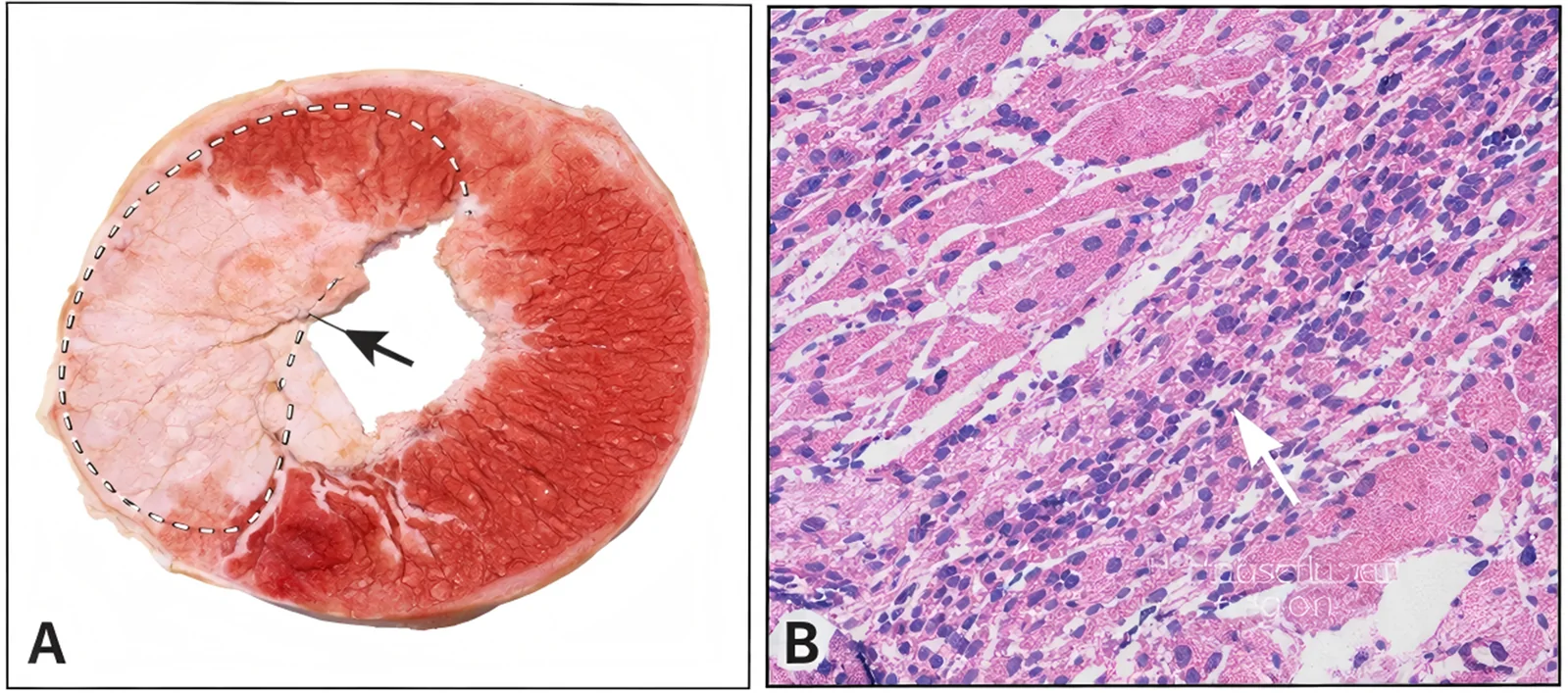
Gross and histopathological assessment of myocardial infarction in the left ventricular myocardium. **(A)** Triphenyltetrazolium chloride (TTC) staining of a transverse left ventricular slice showing pale infarcted myocardium (outlined by dashed line or arrow) and viable myocardium stained red. **(B)** Hematoxylin and eosin (H&E) staining of infarcted myocardium demonstrating myocardial fiber disruption, nuclear pyknosis, and inflammatory cell infiltration (×200).

### 3.5 Comparison of Bcl-2, Bax, caspase-3, and iodine concentrations between normal and infarcted myocardium

Compared with normal myocardium, infarcted myocardium showed significantly lower Bcl-2 expression and significantly higher Bax and caspase-3 expression, indicating enhanced apoptosis in the infarcted region. In addition, iodine concentration was markedly reduced in infarcted myocardium, consistent with decreased myocardial perfusion. These findings demonstrated concordance between apoptosis-related biochemical injury and DSCT-MPI perfusion abnormalities. On this basis, ROC analysis was further performed to evaluate the diagnostic performance of iodine concentration for identifying infarcted myocardium.

#### 3.5.1 ROC curve of iodine concentration in infarcted myocardium by DSCT scanning

ROC analysis was performed using 39 infarcted and 66 normal myocardial segments, whereas the 14 non-infarcted hypoperfused segments consistent with distal MVO were excluded from this binary analysis. At the myocardial segment level, the optimal iodine concentration cutoff was 1.582 mg/mL. At this cutoff, the sensitivity was 89.7% and the specificity was 90.9%. The AUC was 0.848 (95% CI, 0.736–0.959) (Figure 5). The 95% confidence interval had a total width of 0.223 and a half-width of approximately 0.112, indicating limited precision of the AUC estimate. Thus, although the point estimate suggested good discrimination, the lower confidence limit remained compatible with only moderate discriminatory performance.

**Figure 5:**
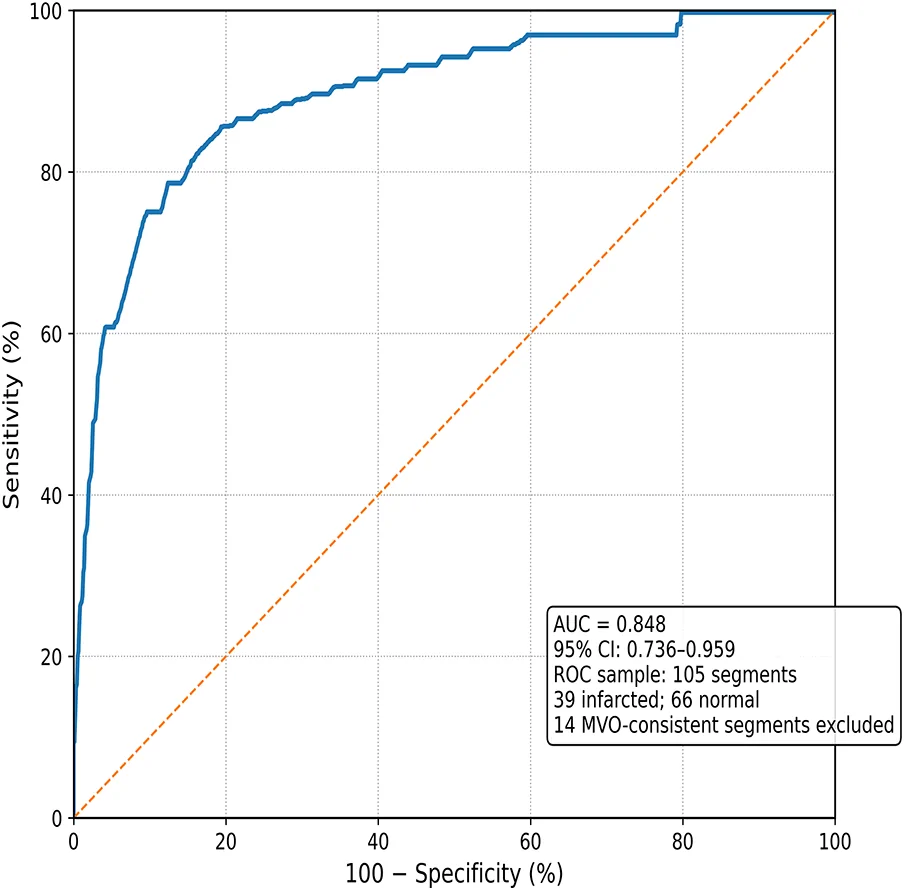
Receiver operating characteristic curve of myocardial iodine concentration for detecting infarcted myocardial segments by DSCT-MPI. The segment-level ROC analysis included 39 infarcted and 66 normal myocardial segments. Fourteen non-infarcted hypoperfused segments consistent with distal microvascular obstruction were excluded from the binary ROC analysis.

### 3.6 DSCT-MPI accuracy analysis

Myocardial segmentation was performed according to the 17-segment model proposed by the American Heart Association (12). Across seven goats, a total of 119 myocardial segments were evaluated, comprising 39 infarcted segments, 14 non-infarcted hypoperfused segments consistent with distal MVO, and 66 normal segments. The binary diagnostic-performance analysis included the 39 infarcted and 66 normal segments, for a total of 105 segments. The 14 MVO-consistent segments were excluded from this analysis and were evaluated separately. DSCT-MPI correctly identified 35 infarcted segments and 60 normal segments, with 6 false-positive and 4 false-negative segments. The resulting sensitivity, specificity, positive predictive value, negative predictive value, and overall accuracy were 89.7%, 90.9%, 85.4%, 93.8%, and 90.5%, respectively. Agreement between DSCT-MPI and histopathological classification was substantial (*κ* = 0.798) (Table 2). Because myocardial segments from the same goat were clustered, within-animal correlation was considered in the statistical analyses as described in the Methods. The distribution of infarcted segments detected by DSCT-MPI closely matched pathological findings, particularly in the lateral and inferior walls.

**Table 2:** Diagnostic performance of DSCT-MPI for detecting infarcted myocardial segments.

Modality	True positive	True negative	False positive	False negative	Sensitivity	Specificity	PPV	NPV	Accuracy	Kappa
DSCT-MPI	35	60	6	4	89.70%	90.90%	85.40%	93.80%	90.50%	0.798

The analysis included 39 infarcted and 66 normal myocardial segments. Fourteen non-infarcted hypoperfused segments consistent with distal MVO were excluded from the binary diagnostic-performance analysis.

### 3.7 DSCT-MPI findings based on myocardial segmentation

A total of 119 myocardial segments were evaluated. Based on the combined DSCT-MPI and histopathological findings, the segments were classified as 39 infarcted segments, 14 non-infarcted hypoperfused segments consistent with distal MVO, and 66 normal segments; no segment was categorized as suspected CMD. Only the 39 infarcted and 66 normal segments, totaling 105 segments, were included in the binary ROC and diagnostic-performance analyses. The 14 non-infarcted hypoperfused segments were analyzed descriptively as a separate MVO-consistent category. Infarcted segments exhibited hypoperfusion on DSCT-MPI with corresponding necrosis on TTC staining. Non-infarcted hypoperfused segments showed significant hypoperfusion (>20% reduction in iodine concentration) without TTC-confirmed necrosis. Normal segments demonstrated neither perfusion defects nor infarction. The proportion of non-infarcted hypoperfused segments consistent with distal MVO was 11.8% (Figure 6).

**Figure 6:**
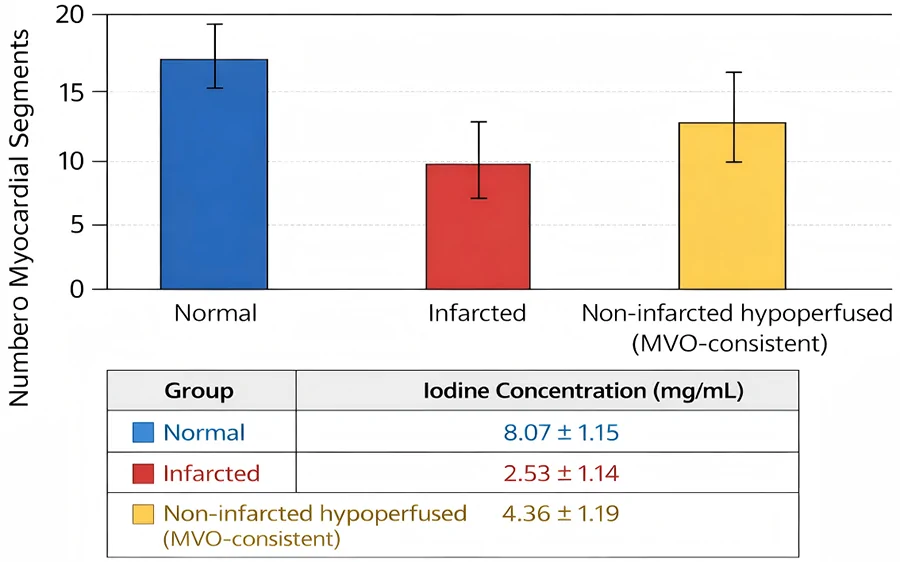
Classification of myocardial segments based on DSCT-MPI and histopathological findings. Myocardial segments were categorized as normal, infarcted, or non-infarcted hypoperfused segments consistent with distal microvascular obstruction (MVO); no segment was classified as suspected CMD. Bars represent the number of myocardial segments in each category, with error bars indicating standard deviation.

### 3.8 Comparison of iodine concentration between different segments

The mean iodine concentration in normal myocardial segments was 2.12 ± 0.22 mg/mL. Infarcted segments showed a significantly lower iodine concentration 1.37 ± 0.19 mg/mL, *P* < 0.001 vs. normal). Non-infarcted hypoperfused segments consistent with distal MVO also demonstrated reduced iodine concentration (1.69 ± 0.17 mg/mL, *P* < 0.001 vs. normal), which was higher than that of infarcted segments (*P* < 0.01) (Figure 7).

**Figure 7:**
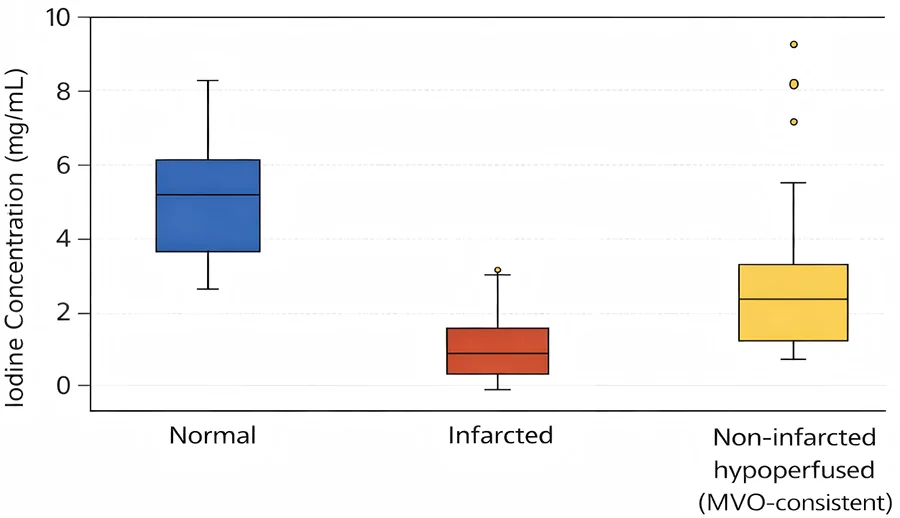
Distribution of iodine concentration across normal, infarcted, and non-infarcted hypoperfused (MVO-consistent) myocardial segments. Box-and-whisker plots showing iodine concentration in normal, infarcted, and non-infarcted hypoperfused (MVO-consistent) myocardial segments. Boxes represent the interquartile range, horizontal lines indicate medians, whiskers denote 1.5×IQR, and dots represent outliers.

Pairwise comparisons using paired t-tests (with goat as clustering variable) further quantified these differences: infarcted vs. normal (t = −20.35, *p* = 1.2 × 10⁻^3^⁰); MVO-consistent vs. normal (t = −10.23, *p* = 3.1 × 10⁻^12^); and infarcted vs. MVO-consistent (t = 8.57, *p* = 4.7 × 10⁻⁸). These results confirm that DSCT-MPI-derived iodine concentration effectively distinguishes among normal, infarcted, and MVO-consistent myocardium, with the latter showing intermediate hypoperfusion that is significant but less severe than in infarcted tissue.

## 4 Discussion

This study established a reproducible large-animal model of myocardial ischemia–reperfusion injury complicated by distal MVO and demonstrated that DSCT-MPI can identify infarcted myocardium and non-infarcted hypoperfused regions with good concordance to histopathological findings. At the myocardial segment level, DSCT-MPI differentiated infarcted segments from non-infarcted hypoperfused segments consistent with distal MVO, reflecting post-reperfusion perfusion heterogeneity rather than evidence of isolated CMD.

MIRI remains a major clinical challenge despite successful epicardial revascularization. Among the mechanisms contributing to impaired myocardial recovery, MVO represents a structural obstruction of the coronary microvasculature caused by distal embolization, endothelial injury, inflammatory responses, and capillary destruction during reperfusion (5, 13, 14). Importantly, MVO is pathophysiologically distinct from CMD, which is characterized by functional or structural abnormalities of the microcirculation in the absence of obstructive coronary artery disease (15, 16). The present study did not aim to diagnose CMD, CMD was not directly evaluated, and no imaging result in this manuscript should be interpreted as a surrogate marker of suspected CMD.

The experimental model combined transient balloon-induced ischemia with distal microsphere embolization to simulate embolization-related MVO occurring after percutaneous coronary intervention. This interventional approach differs from traditional thoracotomy-based coronary ligation models, which primarily induce infarction but do not adequately reproduce microcirculatory embolization associated with clinical revascularization procedures (13, 14). Among large-animal models, dogs, pigs, and goats each offer distinct advantages and limitations. Dogs have historically been used extensively in cardiovascular research, particularly in arrhythmia, heart failure, and device-related studies, and remain useful in selected comparative settings because of their size and electrophysiological similarities to humans (17). Pigs provide one of the most reliable models for myocardial ischemia–reperfusion injury because their coronary anatomy closely resembles that of humans and their native coronary collateral circulation is sparse, so acute coronary occlusion usually produces severe ischemia and infarction in a reproducible manner (18–20). However, the rapid body weight gain of domestic pigs may hinder long-term follow-up studies, whereas minipigs may partially mitigate this issue but differ from domestic pigs in body composition and metabolic characteristics (21). Goats are attractive because of their manageable body size, relatively low maintenance cost, and historically reported sparse intrinsic coronary collateral network (22). Nevertheless, goats may develop extramyocardial collateral flow from skeletal muscle to myocardium, and such collateral circulation can be augmented experimentally, for example by chronic stimulation of the latissimus dorsi muscle (23, 24). Therefore, the goat model used in the present study should be viewed as a practical and reproducible compromise for interventional ischemia–reperfusion research rather than as a perfect surrogate for all aspects of human coronary pathophysiology. Selection of the left circumflex artery allowed controlled induction of regional ischemia while avoiding extensive lethal infarction, thereby improving survival and reproducibility (15, 16).

In humans, the presence and functional significance of coronary collaterals vary substantially across individuals and are influenced by age, severity and chronicity of coronary stenosis, and prior exposure to ischemia. In general, older patients and those with gradually progressive ischemia may develop more extensive collateral networks, although marked interindividual variability remains (25–27). This should be considered when extrapolating collateral-related findings from animal models to clinical myocardial ischemia–reperfusion injury.

Myocardial perfusion imaging techniques, including stress echocardiography, CMR, single-photon emission computed tomography, positron emission tomography, and computed tomography-based approaches, are widely used to assess myocardial perfusion abnormalities (28). DSCT-MPI provides a quantitative assessment of myocardial iodine distribution, which reflects myocardial blood volume and regional perfusion status (29). Compared with CMR, DSCT-MPI offers higher spatial resolution and rapid image acquisition, and it can be integrated with coronary CT angiography in a single examination, which may increase its practical value for combined anatomical and perfusion assessment (29, 30). However, unlike CMR, DSCT-MPI involves ionizing radiation and iodinated contrast administration. In addition, although iodinated contrast and gadolinium share broadly similar extracellular distribution characteristics for delayed enhancement assessment, their enhancement kinetics and acquisition strategies are not identical; therefore, DSCT-MPI and CMR should be regarded as complementary rather than interchangeable techniques (30). CMR remains the reference noninvasive modality for myocardial tissue characterization and microvascular obstruction assessment because it provides multiparametric evaluation without radiation exposure, whereas DSCT-MPI may be advantageous when rapid acquisition, higher spatial resolution, or simultaneous coronary anatomical assessment is desired. In the present study, infarcted segments exhibited marked reductions in iodine concentration with corresponding histopathological necrosis, whereas non-infarcted hypoperfused segments showed reduced iodine concentration without TTC-confirmed infarction, consistent with distal MVO or peri-infarct border zones rather than isolated CMD. Imaging–pathology concordance was high, and segment-based ROC analysis demonstrated good discriminatory performance of iodine concentration for infarct detection (30). These findings support the descriptive utility of DSCT-MPI for assessing myocardial perfusion abnormalities following embolization-induced reperfusion injury, but they should not be interpreted as demonstrating equivalence or superiority to CMR, because no direct head-to-head comparison was performed in the present study. No inference was made that embolization reduced infarct severity.

Histopathological examination confirmed myocardial necrosis, inflammatory infiltration, and microsphere embolization in infarcted regions. Apoptosis-related marker analysis demonstrated decreased Bcl-2 expression and increased Bax and caspase-3 levels in infarcted myocardium, consistent with established molecular responses to ischemia–reperfusion injury (31, 32). These findings further support concordance between imaging abnormalities and tissue-level injury but do not establish mechanistic causality.

Several limitations should be acknowledged. First, CMD was not directly measured, as no assessment of coronary flow reserve, endothelial function, or vasoreactivity was performed. Second, DSCT-MPI was performed at a single time point immediately after model establishment. Microvascular obstruction and peri-infarct perfusion abnormalities may evolve dynamically after reperfusion due to edema, inflammation, and partial restoration of microvascular flow. The acute imaging may have captured a period of relatively pronounced perfusion impairment, potentially overestimating the burden of persistent distal MVO, while missing subsequent expansion, resolution, or redistribution of perfusion defects. Future studies should include serial DSCT-MPI examinations at 24–72 h and, if feasible, at ∼7 days to characterize the dynamic evolution of MVO. Third, the iodine concentration threshold of 1.582 mg/mL was derived and evaluated within the same exploratory dataset and may be optimistically estimated. Its sensitivity–specificity balance should be confirmed in an independent cohort, and it should not be interpreted as a definitive clinical reference value. Fourth, although CMR remains an established reference noninvasive imaging modality for myocardial tissue characterization and MVO assessment, no simultaneous or sequential CMR examination was performed in this study. DSCT-MPI may offer complementary advantages when rapid image acquisition is required, when concurrent coronary CT angiography is desired, or when animals have non-MR-conditional implanted devices. CMR generally requires longer acquisition times and more demanding cardiorespiratory monitoring in anesthetized large animals. Fifth, the 20-µm monodisperse polystyrene microspheres used in this study represent an idealized model of distal microvascular obstruction. Their uniform size and composition do not replicate the heterogeneous clinical embolic material encountered during PCI, which may include fibrin, platelets, erythrocytes, and lipid-rich plaque. This homogeneity may have contributed to the relatively high imaging–pathology agreement observed. Future studies should explore autologous thrombus or heterogeneous embolic mixtures to better simulate clinical microvascular obstruction. Sixth, the number of analyzable animals was small (*n* = 7). Although the segment-level analyses accounted for within-animal correlation using a repeated-measures framework, the effective independent sample size remained determined primarily by the number of animals rather than by the number of myocardial segments. The segment-level AUC was 0.848, but its 95% confidence interval was relatively wide (0.736–0.959), with a total width of 0.223 and a half-width of approximately 0.112. A conventional *post-hoc* power calculation based on the total number of segments was not considered appropriate because it would treat correlated segments from the same animal as independent observations and could consequently overestimate statistical power. Using the observed confidence-interval width as a precision-based approximation, approximately 16 analyzable animals would be required to reduce the expected 95% confidence-interval half-width from approximately 0.112 to 0.075, assuming a similar AUC, segment distribution, and within-animal correlation. Allowing for the observed procedural mortality of one of eight animals, recruitment of approximately 18–19 animals may be required to obtain at least 16 analyzable animals in a future confirmatory study. Finally, echocardiographic assessment of regional wall motion and left ventricular function was not performed before or after model establishment. Although functional assessment was not a prespecified primary endpoint, its inclusion would have strengthened the physiological interpretation of the imaging and pathological findings and is recommended for future studies.

In summary, this study demonstrates that a goat model of embolization-induced myocardial ischemia–reperfusion injury is feasible and reproducible, and that DSCT-MPI can descriptively identify infarcted myocardium and non-infarcted hypoperfused regions consistent with distal microvascular obstruction. The findings support imaging–pathology concordance and provide a translational platform for future investigations of post-reperfusion microvascular injury.

## 5 Conclusion

A minimally invasive and reproducible goat model of myocardial ischemia–reperfusion injury complicated by distal microvascular embolization was successfully established. This model reproduces embolization-related MVO occurring after reperfusion and should not be interpreted as a model of isolated CMD. DSCT-MPI detected regional myocardial perfusion heterogeneity and demonstrated good concordance with histopathological findings.

The ability of DSCT-MPI to identify both infarcted myocardium and non-infarcted hypoperfused regions consistent with distal MVO supports its descriptive utility for assessing perfusion abnormalities in this experimental setting. These findings are limited to model validation and imaging–pathology concordance and do not extend to clinical diagnosis or therapeutic prediction.

## Data Availability

The original contributions presented in the study are included in the article/Supplementary Material, further inquiries can be directed to the corresponding authors.
